# Psychometric properties of the 12-item WHODAS applied through phone survey: an experience in PERSIAN Traffic Cohort

**DOI:** 10.1186/s12955-022-02013-w

**Published:** 2022-07-09

**Authors:** Nasrin Shahedifar, Homayoun Sadeghi-Bazargani, Mohammad Asghari-Jafarabadi, Mostafa Farahbakhsh, Shahrzad Bazargan-Hejazi

**Affiliations:** 1 Cabrini Research, Cabrini Health, VIC, 3144 Australia; 2 Road Traffic Injury Research Center, Tabriz University of Medical Sciences, Tabriz, 5167846311 East Azerbaijan Islamic Republic of Iran; 3 Injury Epidemiology and Prevention Research Group, Turku Brain Injury Center, Turku University Hospital and University of Turku, Turku, Finland; 4 Research Center of Psychiatry and Behavioural Sciences, Tabriz University of Medical Sciences, Tabriz, Islamic Republic of Iran; 5 College of Medicine, Charles Drew University of Medicine and Science, Los Angeles, CA USA; 6 School of Public Health and Preventative Medicine, Faculty of Medicine, Nursing and Health Sciences, Monash University, VIC, 3800 Australia

**Keywords:** WHODAS, Disability, Post-crash, Psychometrics, Road traffic injury, Post injury, Cohort study

## Abstract

**Background:**

Due to limited capability to function in post-injury daily life injury, survivors need to be reliably assessed without need to commute more than necessary. The key action is to determine the level of functioning difficulties. Having the opportunity of conducting a national post-crash traffic safety and health cohort study, we aimed to translate into Persian and assess the psychometric properties of the World Health Organization Disability Assessment Schedule 2.0 (WHODAS 2.0) through phone surveys six month post injury.

**Methods:**

First, having World Health Organization permission, we tested the translation validity by forward translation, expert panel evaluation, back-translation, pre-testing and cognitive interviewing, and finalizing the Persian WHODAS. Then, through a psychometric study within a national cohort platform, the validity, reliability and applicability of the 12-item WHODAS was assessed through phone surveys. We included data of 255 road traffic injury patients enrolled from the cohort at six-month follow-up. The psychometric assessment (internal consistency reliability and stability reliability) was conducted on test–retest data of 50 patients with an average 7-day time span. An exploratory factor analysis tested the construct validity using extraction method of principal component factor and oblique rotation on data from 255 patients. Regarding the multiple criteria including an eigenvalue > 0.9, Cattell’s scree test, cumulative variance, and the theoretical basis, the minimum number of factors were retained. Data were analyzed using STATA statistical software package.

**Results:**

The respondents were mostly male (81%), employed (71%), educated (87%), and with a mean age of 37.7(14.9). The Persian version had high internal consistency reliability (Cronbach’s α = 0.93) and excellent stability reliability (ICC = 0.97, 95% CI: 0.92–0.98). An exploratory factor analysis retained four factors defining 86% of all the variance. Factors of Self-care, Mobility, and Cognition were completely retained.

**Conclusions:**

The brief Pesrian WHODAS 2.0 was highly reliable and valid to be applied through phone interviews post injury.

## Introduction

Road traffic crashes (RTCs) result in 1.35 million deaths annually and leave 20 to 50 million people with non-fatal injuries, with many suffering a disability [1]. The injuries account for 41.2 million years of healthy life lost [2–4], and 90% of disability-adjusted life years in Low and Middle Income Countries [5]. In Iran, road traffic injuries (RTIs) are the second leading cause of death [6, 7], and the third cause of disability adjusted life years [8]. Over 2600 person years of life lost are attributed to RTIs [9]. The people hospitalized due to RTIs mainly suffer severe injuries resulting in short- or long-term difficulties in functioning [10]. Only with the functioning information would we recognise their right health care needs [11, 12].

The concepts “functioning” and “disability” in public health and clinical settings represent information on how the population accomplishes its activities and participation [13]. Functioning is “generic term which includes body functions and structures, activities and participation. It indicates the positive aspects of the interaction between the individual (with a health condition) and its contextual factors (personal and environmental factors)” [14]. Disability is an umbrella term for deficiencies of body functions, activity limitations or participation restrictions [13]. The controversial issue is how to measure the functioning level, and many tools have been developed. however the tool by the World Health Organization Disability Assessment Schedule 2.0 (WHODAS 2.0) was developed on an inclusive categories of International Classification of Functioning, Disability and Health (ICF) [14]. Although there are many tools developed to measure functioning, WHODAS 2.0 is reputable for several chief characteristics. For instance, the 11^th^ International Classification of Disease–ICD-11 added the section “V Supplementary section for functioning assessment” to form functioning profiles and overall functioning scores [15, 16].

Tool’s psychometric properties may be affected through the method of data collection such as phone surveys comparing with in-person interviews. So it is critical to assess the tool’s psychometric properties for various data collection. Then, very little similar work has been done so far. One of the major field is assessing disability in injuries post-crash and post discharge. For such assessing, there is rare opportunity to have in-person visits, in some settings particularly in low and middle income countries (LMICs) and then the resources do not support the researchers to do in-person interviews. It is critical to have robust tools to use for phone surveys. To assess this, we had a great opportunity of a cohort study. This setting helps to apply the tool and assess its psychometric properties through national cohort platform of Post-crash Traffic Safety and Health Cohort Study, Prospective Epidemiological Research Study in IrAN (PERSIAN) [17, 18]. Then the key action was to apply reliable and valid tools in measuring the outcomes of RTIs such as functioning difficulties, a common consequence. So, the expert team chose the inclusive tool of WHODAS 2.0 to be administered to specifically RTI survivors through phone, with great care, sensitivity, and searching.

Although the available translations are neither specified for the target group with road traffic injuries, nor applied for phone-based surveys [19–22], the academic expert panel meticulously reviewed and piloted it among road traffic injury patients in the setting of the prospective PERSIAN Traffic Safety and Health Cohort Study. As its validity was not approved in the setting, we communicated with WHO permissions team and obtained its permission to retranslate the original version to apply in the PERSIAN Traffic Cohort Study (ID: 380480). As the short form of this globally approved tool has a good explanation of the full-version tool and takes five minutes on average, as well as considering the physical health conditions of RTIs interviewees, we utilized and psychometrically assessed the 12-item WHODAS 2.0, through phone interviews six months post injury. Also, to our knowledge, there are not published psychometric works in which the short-version tool was used in phone surveys and in RTI patients in the world, except for one phone survey among critically-ill patients admitted to intensive care units, and one interview-based study among patients with road traffic injuries. The paucity of such all-inclusive tool on creating the functioning profile of people involved in RTCs is sensible reason to conduct the translation, and to examine internal consistency, reproducibility, time consistency, and construct validity of brief WHODAS 2.0 among study population through phone surveys six months post injury. Then, it would pave the way for study of the functioning problems caused by road traffic injuries. We expected that the short version would demonstrate excellent internal consistency and test–retest reliability, defined as values of 0.80 or greater and 0.90 or greater, respectively.

## Materials and methods

### Phase one: translation of the 12-item WHODAS 2.0

The World Health Organization (WHO) permitted the Road Traffic Injury Research Center (RTIRC) to translate the 12-item WHODAS 2.0 into Persian language for use specially in the context of the Traffic Safety and Health Cohort Study (permission ID: 380480). Our expert team composed of members from RTIRC, and physical medicine and rehabilitation Research Center, Tabriz University of Medical Sciences. It conducted an iterative, rigid, and meticulous process of translation, according to the translation guideline recommended by the WHO in five steps: (1) forward translation; (2) Expert panel; (3) Back-translation; (4) Pre-testing and cognitive interviewing, and (5) Final version [23, 24].

### Phase two: assessment of reliability and factor structure

The psychometric properties of the brief WHODAS 2.0 was evaluated at six months after crash through phone-based interviews. This study was conducted in a clinical population. The source population of the study were RTIs survivors enrolled from post-crash traffic safety and health cohort study [18]. All participants hospitalized in one of two referral trauma centers, Shohada and Imam Reza, in Tabriz, were followed up through the cohort study at six months after crash. We conducted on a representative sample of RTIs survivors 18 years and above, between 21 May 2020 and 20 March 2021. The whole hospitalized patients have been recorded in the database of Integrated Road Traffic Injury Registry System (IRTIRS) since 2019 [25]. To examine the reliability of the instrument, the minimum number of sample required was 50 for detecting the value of 0.4 for ICC, with alpha and power defined at 0.05 and lower than 90%, respectively [26]. In order to conduct factor analysis, a maximum number of 21 participants per item was taken to attain the best model fit [27]. So, 255 patients were included in our sample. The selected participants were replaced by the same sex and age if they refused to participate. As inclusion criteria, participants must have been hospitalized due to RTIs six months prior to their interview date for at least 24 h, registered, aged 18 or over, and informed consent was required. People unable to complete the questionnaire due to any disorders were excluded from the study. The study was carried out by an appropriately trained interviewer with broad experience (≥ 4 years) routinely collects data from registered patients on phone calls [17]. The completeness of tools was mostly guaranteed since the interviewer filled them out.

### Measures

#### The 12-item WHODAS 2.0

As a general measure, WHODAS 2.0 was developed based on an inclusive set of categories considered in the framework of ICF to measure disability during the preceding 30 days [14]. It can be used for epidemiological studies and other purposes. This questionnaire was researched by the WHO in many countries, where its reliability, convergent validity with other assessment instruments, constant factor structure, and other psychometric properties were analysed [28].

The short version describes 81% of the variance of the full version. Researchers can compute overall functioning scores, administer through an interview during the mean interview time of about 5 min [29]. The response choices for each item ranged on an ordinal scale, from zero denoting “none” to four denoting “extreme/cannot do”. The individual scores in each subscale were estimated by simply adding up the results of the two relevant items [30]. Each subscale contains two questions from the corresponding subscale in the full version: Subscale 1: Cognition (items 3 (learning) and 6 (concentrating) of the questionnaire); Subscale 2: Mobility (items 1 (standing) and 7 (walking)); Subscale 3: Self-care (items 8 (washing) and 9 (getting dressed)); Subscale 4: Getting along with people (items 10 (dealing with people) and 11 (maintaining a friendship)); Subscale 5: Life activities (items 12 (day-to-day work) and 2 (household responsibilities)); Subscale 6: Participation (items 4 (community activities) and 5 (emotions)) [24, 31]. A simple way of calculating the results was used for the psychometric evaluation of the tool. The total score is a percentage as follows:

$${\text{Total}}\;{\text{score}} = \left( {\sum {\text{item}}\;{\text{scores}}/48} \right) \times 100$$

It ranged from 0 (no disability) to 48 (complete disability). A higher result indicates a higher level of disability [29]. To draw a comparison, raw total scores and subscale scores were then converted into a 0–100 scale as normalized scores, using the complex method of scoring [32]. We categorized the total disability scores according to the ICF severity ranges (no problem, 0–4; mild disability, 5–24; moderate disability, 25–49; severe/extreme disability, 50–100). We considered a disability score of greater than or equal to 25 to indicate ‘disability’ based on the WHODAS ICF [33]. When only one item from the 12 items was missing, the average of the remaining 11 items was assigned to the missing item. If more than one item was missing, the survey was rejected [34].

### Socio-demographic data

Basic socio-demographic data (sex, age, marital status, education, and job) and selected crash-related information [35] (used vehicle, counterpart vehicle, crash mechanism, and injured person’s role) were collected.

### Statistical analysis

Initial analyses of the WHODAS and baseline characteristics data were performed using descriptive statistics. First an inter-item correlation matrix was visually observed. Correlations were defined as excellent (> 0.75), moderate (0.50–0.74), fair (0.25–0.49), and no meaningful correlation (< 0.25) [36]. With a sample size of 255 patients, this study has 80% power (two-sided P = 0.05) to detect a correlation coefficient of 0.2 or greater. The data were analyzed using STATA statistical package version 15 (StataCorp LLC, Texas) and adopted a significance level of 0.05.

### Scale score reliability

#### Internal consistency reliability

Internal consistency indicates the extent to which the items in a subscale are correlated. The homogeneity was evaluated using Cronbach’s Alpha statistic [37]. A value between 0.5 and 0.75 was considered moderate while values between 0.75 and 0.9 indicate good and values greater than 0.90 specify excellent reliability [38].

#### Test–retest reliability

Another measurement used to directly assess the scale score reliability of the tool was repeatability assessment using the test–retest method. Comparing the results of the first and second measurements examined the consistency over time. A group of 50 people was interviewed on telephone, between 21 May 2020 and 15 June 2020. All of them participated in both test and retest measurements. They did not have any treatment during that week period or changes in their medication. The average time between two measurements was 7 days (range from 6 to 8 days). Generally, a one-week time frame is applied on functional measurements [39]. In this study, inconsistencies might be produced by change in functional conditions of patients (physical and mental conditions) [40]. Moreover, the minimum detectable consistency is reachable by 7-day time interval with regard to lessen the potential for learning effect [41]. Other sources of inconsistencies such as dissimilarities in testing procedure and testers were not true as we took consistency, fixed protocol for testing, and one tester. Along with the criteria of the study, specific exclusion criteria for this step included having psychiatric problems affect the validity of the responses or an event experienced during the test–retest interval significantly increases the level of disability (e.g. disease, another car crash, and fall).

The normality of total scale and subscales’ distributions were tested based on the value of skewness and kurtosis. The test–retest correlation was measured by tau Kendal b. The reliability of the method was assessed by intra-class correlation coefficient (ICC). Scores of test–retest were analysed using the “single-rater type (k = 1), absolute-agreement, 2-way mixed-effects model to produce the intra-class correlation with 95% confidence intervals [38]. An ICC coefficient of ≥ 0.75 was considered as evidence of measurement stability. ICC between 0.4 and 0.75 indicates fair to good reliability, and ICC < 0.4 indicates poor reliability [42].

### Factor structure

#### Exploratory factor analysis (EFA)

The exploratory factor analysis is a fundamental tool to define the optimal count of latent variables in the validation of 12-item WHODAS 2.0 completed by 255 patients six months after crash on phone calls. As the correlation matrix was not compound symmetric, we did not run the method of Maximum Likelihood. Since 5-point Likert scales, are subject to lack of normality, we extracted factors using method of principal component factor and oblique rotation on data of 255 patients, from May 2020 to March 2021. To test the assumptions of EFA, the overall KMO (Kaiser–Meyer–Olkin) to measure sampling adequacy was 0.81, and the.

Bartlett’s test of sphericity proved an appropriate model (X^2^ = 4278.422 and P < 0.0001) [43].

**Fig. 1:**
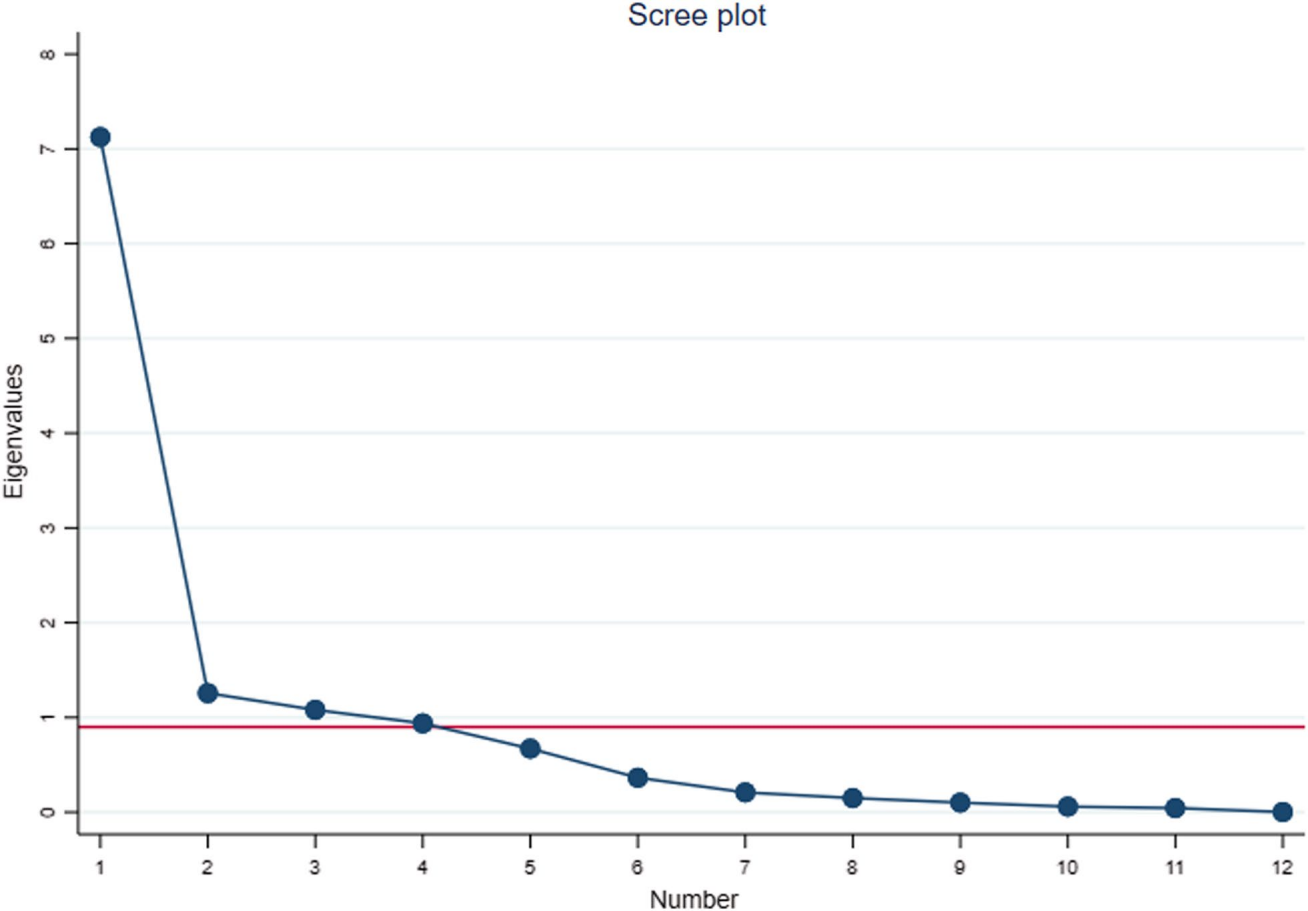
Scree plot of eigenvalues for factor retention

In the EFA, it is aimed to explain the maximum variance using a small number of factors. It is essential to apply multiple criteria and wisely judge each acceptable solution to retain the most appropriate factor numbers. In fact, no single method has been created to be correct in all conditions [43]. Moreover, it is not recommended to use Kaiser-Guttman method of “eigenvalue 1” anymore and the researchers could act with more flexibility of preferred cut-off when applying multiple criteria [43–45]. Then, we used multiple criteria of eigenvalue, Cattell’s scree plot, cumulative variance, and theory, to determine the number of factors (Fig. 1).

### Floor and ceiling effects

The floor and ceiling effects were derived from the percentage of respondents with the lowest and highest possible scale scores for individual items of the tool. Ceiling and floor effects were considered present when it is higher than 15% [46].

## Results

The research data were data for the brief-version WHODAS 2.0, and crash-related and socio-demographic characteristics. A total of 255 subjects were approached by a research assistant. The majority of participants were male (81%). The mean age (SD) was 37.7(14.9; 18–86 yrs). The respondents were mostly employed (71%) and educated (87%) (Table 1). The mechanism of half of the crashes was vehicle-vehicle collision. The majority of subjects were motorcycle and car drivers. A fourth of patients were passenger or pillion passenger when they had crash (Table 1).

**Table 1:** Baseline characteristics of study population (N = 255)

Variables	Categories	N = 255 (%)
Sex	Male	207 (81%)
	Female	48 (19%)
Age	18–24 yrs	54 (21%)
	25–44 yrs	124 (49%)
	45–64 yrs	59 (23%)
	65 yrs and above	18 (7%)
Marital status	Never married	77 (30%)
	Married	165 (65%)
	Divorced/widowed	13 (5%)
Education level	Illiterate	33 (13%)
	School education	180 (71%)
	Academic education	42 (16%)
Job	Employed	182 (71%)
	Unemployed	73 (29%)
Injured person’s role	Driver	130 (50.9%)
	Pedestrian	55 (21.6%)
	Passenger/pillion passenger	69 (27.1%)
	Undefined	1 (0.4)

The average disability score for the study population (N = 255) was 26.87 ± 22.1 on a scale of 0 to 100 points, with 50 percentile at 25.

### Phase one: translation of the 12-item WHODAS 2.0

The forward translation was done by three qualified translators as native-like English speakers and one of them is a professor living in the USA. They were familiar with health and disability terms. The tool with 15 items and 402 words was translated into Persian language with 15 items and 380 words. Then the panel discussed on the term “emotionally affected” in item 5 (emotions) which takes several Persian meanings, and finalized. An independent linguist blind to the original English terms performed back translation with 15 items and 365 words. Three experts examined compatibility of the forward and back translations per each item, separately on a 4-level Likert scale. Measuring the compatibility, modified Kappa rate (> 0.76) revealed that two items were completely compatible and the others’ compatibilities were acceptable, except for the item 5 (emotions) lower than 0.76, due to the semantic complexity of the used word in forward translation. Following the expert panel and translators’ discussion and minor amendments, the translated questionnaire was completed by several people as a pre-test to examine if the questions were translated and written in a clear and comprehensible manner. The final translation is presented in Table 2.

**Table 2:**
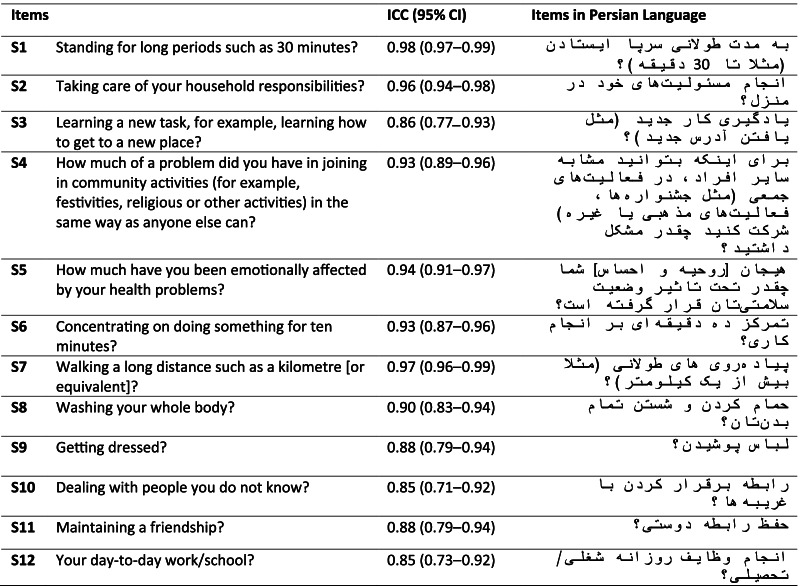
Item reliability of the short form of WHODAS 2.0

### Phase two

#### Factor structure

An exploratory factor analysis was conducted for 12 item WHODAS 2.0. We retained four factors based on multiple criteria. Based on the Cattell’s scree plot, we recognized the point of break at which the shallow scree initiates, then maintained four factors that do not join the scree [47] (Fig. 1). Based on the cumulative percent of variance, the first four factors account for a proportion 0.86 of the total variance. More than 80% of variance was explained by four factors, 1/3 as many factors as variables [48]. Kaiser-Guttman rule allowed us to keep only three factors. Although the fourth factor added 0.14 variance which might seem little, it was theoretically sensible and meaningfully interpretable, too [48]. Then, we retain four factors as stated by criteria of eigenvalue, Cattell’s scree plot, cumulative variance, and theory, which led us to extract factors with eigenvalues above 0.9.

All items showed uniqueness less than 0.2, except for the item 6 for “concentrating doing something for ten minutes” with 0.44 [49]. Accordingly, four factors with factor loadings equal or > 0.3 on 255 people were retained. Items grouping on the same factor presented four factors as follows: factor 1, “social/self-activities” included items involving “Life activities”, “Participation”, and “Getting along” as in the original instrument; factors 2, 3 and 4 indicated activities of “Mobility”, “Self-care”, and “Cognition”, respectively as in the original tool. The loading matrix indicated the item 2 cross loaded onto the factors one and four, though the factor loading was negative for factor four. Each and every item of factors 2 to 4 cross-loaded onto the factor one, too. The highest loadings were considered the most representative, except for the item 6 (Table 3). Since the difference of its loadings was less than 0.1, we categorized it in factor 4 in accordance with the theory of original WHODAS. The total Cronbach’s α was 0.92 ranged from 0.91 for item 5 to 0.93 for both items of Cognition.

**Table 3:** Factor loading matrix for WHODAS 2.0 items (N = 255)

Items (number of item)	Factor1	Factor2	Factor3	Factor4
Maintaining a friendship? (11)	0.9438			
Dealing with people you do not know? (10)	0.9393			
How much of a problem did you have in joining in community activities (for example, festivities, religious or other activities) in the same way as anyone else can? (4)	0.9266			
How much have you been emotionally affected by your health problems? (5)	0.8671			
Your day-to-day work/school? (12)	0.8224			
Taking care of your household responsibilities? (2)	0.6231			−0.5900
Concentrating on doing something for ten minutes? (6)	0.4884			0.3985
Walking a long distance such as a kilometer [or equivalent]? (7)	0.5777	0.7656		
Standing for long periods such as 30 min? (1)	0.6050	0.7171		
Washing your whole body? (8)	0.6102		0.7529	
Getting dressed? (9)	0.6140		0.7500	
Learning a new task, for example, learning how to get to a new place? (3)	0.3865			0.8528
Eigen values	7.12	1.25	1.07	0.93
Cumulative variance (%)	0.59	0.69	0.78	0.86

**Fig. 2:**
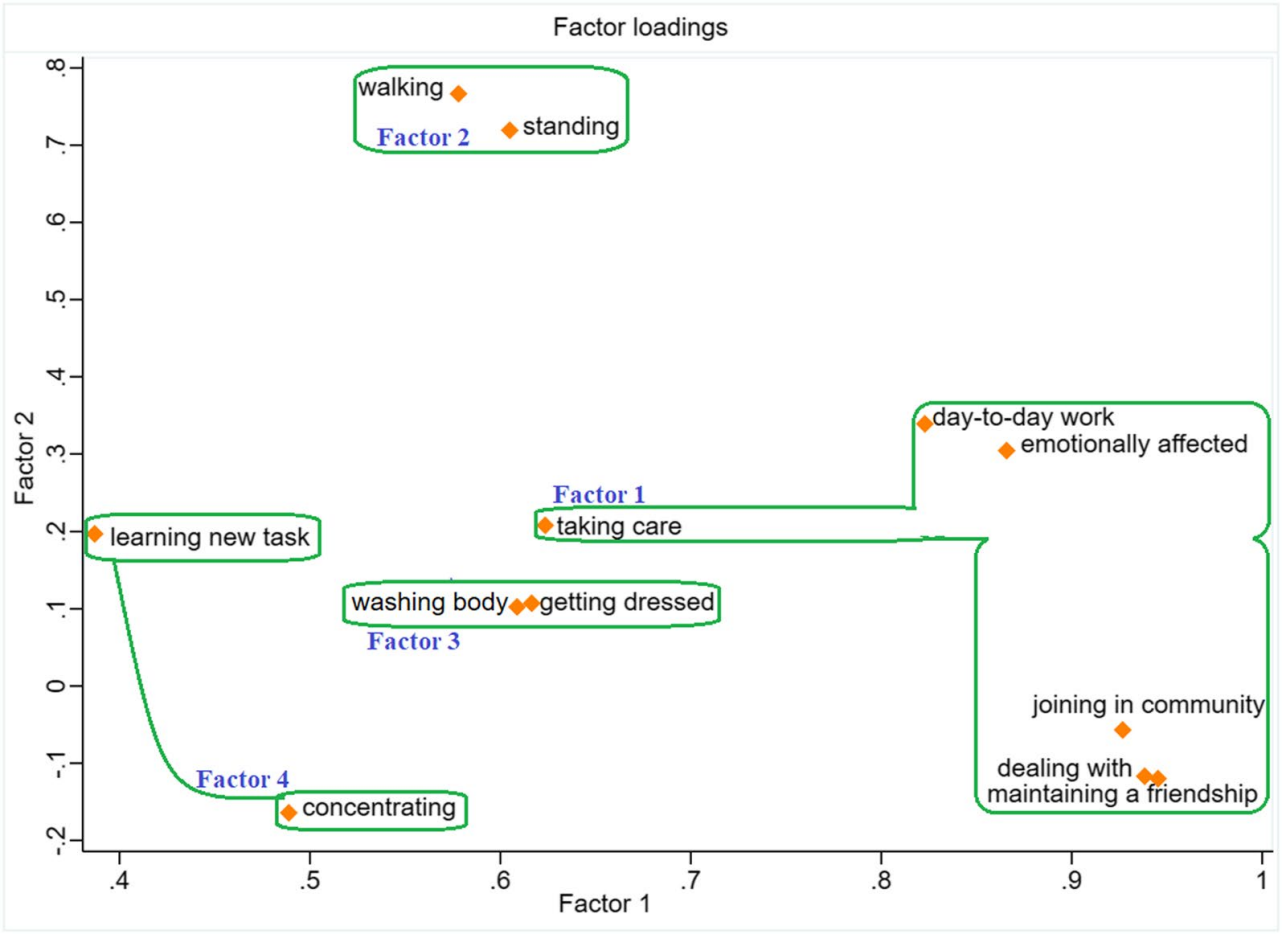
Loading plot of factors, based on principal component factor extraction and oblique rotation

The PCA-based factor loading plot depicted clusters containing items with similar correlations of more than 0.30 with a factor (Fig. 2) [50]. There were three items separated from other clusters (learning, concentrating and household responsibilities), cross loaded onto two factors of one and four.

### Scale score reliability

#### Floor and ceiling effects

For the total data (N = 255), the floor effect values (an answer of “no problem”) ranged from 24% (item 12 (day-to-day work)) to 91% (item 6 (concentrating)). All values were above 25% except for items 5 (emotions) and 12 (day-to-day work). The ceiling effect values (an answer of “extremely large” or “cannot do”) ranged from 0.4% (item 6 (concentrating)) to 6% (item 1 (standing)) except for item 7 (walking) with 32% ceiling effect.

#### Internal consistency

For the total data (N = 255), the Cronbach’s α coefficients resulted in 0.93 (total scale) ranged from 0.92 (items 1 (standing), 4 (community activities), 5 (emotions), 10 (dealing with), 11 (maintaining a friendship), and 12 (day-to-day work)) to 0.93 (items 2 (household responsibilities), 3 (learning), 6 (concentration), 7 (walking), 8 (washing) and 9 (getting dressed)). The Cronbach’s α coefficients for subscales fluctuated between 0.82 (for Participation) and 0.88 (Cognition).

Internal consistency was assessed for the test–retest data (N = 50) by estimating Cronbach’s α coefficient for the whole scale (0.89), subscales, and items ranged from 0.88 for items 8 (washing) and 9 (getting dressed) to 0.90 for item 7 (walking). The Cronbach’s α coefficients for individual subscales were high (Table 4).

**Table 4:** Reliability indices of the Persian version of 12-item WHODAS 2.0 in phone-based survey (N = 50)

Subscales (items)	Mean (SD)		Cronbach’s alpha	ICC (95% CI)	Agreement	Kendall's tau-b^*^
	test	Retest				
Cognition (3 & 6)	3.10 (1.90)	2.86 (1.80)	0.79	0.93 (0.87–0.96)	Excellent	0.71
Mobility (1 & 7)	7.02 (2.69)	7.06 (2.62)	0.84	0.99 (0.98–0.99)	Excellent	0.91
Self-care (8 & 9)	3.64 (2.26)	3.36 (2.07)	0.77	0.92 (0.85–0.95)	Excellent	0.75
Getting along (10 & 11)	5.56 (2.34)	4.94 (2.19)	0.78	0.89 (0.78–0.94)	Good	0.75
Life activities (12 & 2)	6.68 (1.47)	6.50 (1.43)	0.75	0.94 (0.89–0.97)	Excellent	0.87
Participation (4 & 5)	5.90 (1.84)	5.68 (1.74)	0.76	0.96 (0.93–0.98)	Excellent	0.84
Total score	31.90 (9.52)	30.40 (8.68)	0.81	0.97 (0.92–0.98)	Excellent	0.82

Each subscale has two items. *At significance level of 0.0001

#### Test–retest reliability

Consistency over time was examined by comparing the results of the first and second measurements in 50 people. The skewness-kurtosis test showed the normal distribution for some subscales 4, 5, and 6, on test data. Kendall's tau-b indicated no significant differences between test and retest responses (Table 4).

The reliability of the test–retest method was confirmed using the ICC. Time consistency became very high as ranged from 0.89 (subscale 4) to 0.99 (subscale 2) indicating good to excellent reliability and stability. For the overall result, the ICC was 0.97, demonstrating excellent test–retest reliability (Table 4). The ICC for items ranged from 0.85 to 0.97, good to excellent stability (Table 2).

The test data showed the lowest and the highest mean disability score in Cognition (3.1), and Mobility (7.0), respectively (Table 4).

## Discussion

This paper developed the Persian WHODAS 2.0 by translation, then described psychometric properties of the Persian version of phone-based interviewer-administered tool for road traffic injury survivors in Tabriz, Iran, six months after crash, between 21 May 2020 and 20 March 2021. In this study, we translated the tool into Persian, and indicated that Persian WHODAS 2.0 is psychometrically reliable to the great extent in phone-based assessment of disability. Satisfactory internal consistency reveals the Persian WHODAS to measure the construct of disability and deliver high reliable score at item level and total score in our representative sample. The schedule is robust to changes in the same subjects over time and less prone to measurement error caused by time elapse, as ICC showed excellent reliability. Exploratory factor analysis discloses the high validity of the schedule as items hang consistently together to create the single construct of disability. The final model represented four factors of social/self-activities, cognition, mobility, and self-care, accounted for 86% of the total variance.

This study worked on the translation of the brief WHODAS, although, it was translated and validated in different languages even in Persian [20, 22]. However, the Persian version was applied neither specifically through phone survey nor in RTIs patients. The psychometric properties of a questionnaire could be affected through the method of data collection such as phone surveys comparing with in-person/self-administered interviews. The available Persian translation was used in interview surveys [20, 22], not phone surveys. Moreover, in data collection by a questionnaire, there may be misunderstanding of questions and recall bias because of language/cultural barriers. Also, questionnaires are not exactly developed for people with certain conditions [51]. The results may be influenced by subjective measures, too [51]. Because the team planned to apply the tool later in a national prospective post-crash cohort study, it was so critical to have a robust Persian version in informal Persian language and easy to understand. Thus, the team decided to translate the available version. To do so, we obtained the permission from the WHO (permission ID: 380480).

According to the exploratory factor analysis, the Persian WHODAS 2.0 measured disability with four components accounted for 86% of total variance and demonstrated better results compared with a study in trauma patients [22], and some other studies [52–54]. The factor loadings of the individual items were strong, as eleven items’ factor loadings were above 0.60 and one item showed 0.39, consistent with factor loadings in a study [52]. One factor, titled social/self-activities, contains items pertained to the subscales of Getting along, Life activities, and Participation, similar to a study in terms of the items of the extracted factor, not the number of extracted factors in the study of Abedzadeh–kalahroudi [22]. Despite similar number of sample in both studies, her study gathered data by interview with patients categorized in five groups (Traffic, Home, Assault, Work, and Other). The data collection method and uniformity of sample may cause in contradiction and fewer number of extracted factors. Three separate factors certainly distinguished cognition, mobility, and self-care subscales. The structure is quite similar to the structure found in the original English version [29]. A study indicated different groupings, attained a two-factor solution in older people from different dementia sites, with subscales of mobility, life activities, cognition, and participation in its first dominant factor and the self-care and getting along subscales in its second factor [55]. It seems higher number of sample size would help to extract the remaining three subscales separately as the same three distinct factors as in the original version. However, it is better to consider the “most appropriate” rather than the “correct” number of factors.

No measurement difference was indicated between the test and retest results. In spite of importance of using the spectrum of non-extreme in face-to-face interviews, it is not practical in phone-based surveys. However, we attained high consistency between total and subscales’ scores in phone-based surveys.

The Cronbach’s α coefficient for the Persian version indicated the ideal internal consistency and better results compared with a study among Syrian refugees (α = 0.74)[52]. The Cronbach’s α coefficient was similar to several studies with comparable target groups (Ethiopian road traffic patients α = 0.88 [56], in trauma patients α = 0.91[22]). Although both studies used the tool through in-person interviews, not phone interviews, we reached the high internal consistencies as Ustun et al. reported [29]. The result was the same as the result of studies with different target groups (a global study [34], Brazilian study in patients with chikungunya (α = 0.93) [57], in only phone-based psychometric study of WHODAS among critically ill patients (α = 0.91) [36], Spanish version in depressed patients (α = 0.89)[53], and Portuguese version in patients with musculoskeletal pain (α = 0.84) [58]). It is necessary to note that the internal consistency between WHODAS scores of patients with different injuries such as traumatic brain injury and spinal cord injury was high [59, 60]. Then, comparing the results of such different studies could be reasonable. The highest Cronbach’s α coefficient was related to Cognition in consistent with a study in Ethiopia [56], as both worked on RTI patients.

The study confirmed that the scale was consistent over time representing excellent test–retest reliability as ICC fell in a range from 0.89 (Getting along) to 0.99 (Mobility) which is in line with a study in people with and without hand injuries (ICC value of 0.88) [61]. A study in patients with Kashin-Beck disease affirmed a good repeatability of the 12-item WHODAS 2.0 [62]. We reached higher time consistency at total and subscale levels compared with a study in Polish society (ICC value of 0.91, ranged from 0.50 (Self-care) to 0.97 (Mobility)) [24]. Both studies showed the highest ICC for the subscale of Mobility with the items of “Standing for long periods such as 30 min?” and “Walking a long distance such as a kilometre [or equivalent]?” It could be explained by the fact that these activities are of physical activities rarely affected by any actions through the time between test and retest. Generally, the results might differ regarding the mode of administration (in-person interviews versus our phone-based interview) and characteristics of the study population such as age, health status, country of origin) [63]. Moreover, the quality of translation definitely influence the understanding of questions by interviewees.

Data showed floor effects but no ceiling effects, except for the item 7 (walking)). It was almost in line with baseline result of a study in critically ill patients [36] as well as a study of polish society [24]. Overall, study setting and way of using a tool might bring about floor and ceiling effects in data. In this study, the tool was used through phone interviews. However, the content of the tool was examined by asking the opinion of interviewees about the clarity and comprehensibility [64].

This tool is short, and have less complex questions, so it is a proper choice to use in such prospective cohort study with various questionnaires and examinations. The tool was completed via phone calls. This technique is essential for a couple of reasons. It takes around five minutes to complete it on phone calls [65]. It enabled data collection without unnecessary travel of patients to the study site/hospital or travel of interviewers to the place of patients’ residence, due to patients’ movement restrictions. Consecutively it helped to avoid their exposure to pathogens in the current situation of the Pandemic COVID-19. Also, in-person interview is not an easy-to-access opportunity for every researcher/participant. Our data were attained by one assistant interviewer with more than four years of experience in running phone interviews to fill out questionnaires determined in the IRTIRS. So, lack of such skills would also lead to different results in phone-based interviews. Since conducting telephone interviews requires special communication skills, it is necessary to repeat the research on the basis of phone-interview conducted by different interviewers. On the other hand, some patients such as elderly patients and those with hearing problems may have problem with phone survey. So it is recommended to reassess the tool in specific target population such as elderly people.

In this study, subjects were recruited at six months after crash. It is important to ensure that their function was partially stable and patients have got recovered from their minor injuries causing slight physical restrictions [17].

As different works on gender difference in road traffic injuries reported, male users have higher risk of injuries [66–71] due to various reasons such as their higher exposure to driving, patterns of risky traffic behaviours, and so on. The current study also indicated the same pattern, especially the common proportion in Iran.

### Strengths and limitations

To the best of our knowledge, this is the first study in which the psychometric properties of the Persian version of the 12-item WHODAS 2.0 have been evaluated through phone interviews in road traffic injury patients above 18 years in the region and even the region. This study enables assessing functioning, and disability in injured population unable to attend the study site and conducting studies to compare results between different settings.

The age limitation in the tool assessment is defined owing to some feasibility issues. So, the psychometric assessment of brief WHODAS in teenagers is recommended. We plan to extend the research by developing and administrating the tool to the population with Azeri language, the most common language in the northwest Iran. Since it is necessary to use the valid and reliable tool for people whose mother language is Azeri, and there are difficulties in running the Persian version for aged and illiterate people. Regarding the effect of various communication skills, we recommend running phone-based survey by different interviewers.

The potential sources of bias might be considered when making inferences based on our findings. This study as an exploratory cross-sectional one, six months after crash, never provides evidence on the longitudinal performance of the tool for short or long term timing after crash. Moreover, this was a cohort study involving patients with road traffic injuries hospitalized in the only two referral hospitals in Northwest Iran and registered. Thus, it mainly represents the patients needed to be hospitalized post-crash. Conducting the research on the basis of phone interviews in the same and different contexts but in larger sample size is needed. We suggest using a longitudinal design to evaluate responsiveness to change and test–retest reliability.

## Conclusion

The current study translated into Persian and examined the psychometric properties of the 12-item WHODAS 2.0 in a study population experienced road traffic crashes and injuries approximately six months prior to study time and hospitalized in one of two referral trauma centres located in the city of Tabriz, Iran. We realized that the disability assessment tool is short and easy-to-use, and it is suitable to apply in phone-based interviews during follow-ups of injured patients above 18 years.

## Data Availability

Data are available upon reasonable request. The interested researchers may contact Dr. Homayoun Sadeghi-Bazargani (PI). The applications will be reviewed upon approval by the research council and the regional ethics committee of Tabriz University of Medical Sciences. The study website is https://cohortsafety.tbzmed.ac.ir/.

## Abbreviations

WHODAS 2.0: World Health Organization Disability Assessment Schedule 2.0
RTIs: Road traffic injuries
ICF: International Classification of Functioning, Disability and Health
ICD-11: 11Th International Classification of Disease
LMICs: Low and middle income countries
PERSIAN: Prospective Epidemiological Research Study in IrAN
RTIRC: Road Traffic Injury Research Center
IRTIRS: Integrated Road Traffic Injury Registry System
ICC: Intra-class correlation coefficient
EFA: Exploratory factor analysis
